# Faecal microbiota characterisation of horses using 16 rdna barcoded pyrosequencing, and carriage rate of clostridium difficile at hospital admission

**DOI:** 10.1186/s12866-015-0514-5

**Published:** 2015-09-16

**Authors:** Cristina Rodriguez, Bernard Taminiau, Bastien Brévers, Véronique Avesani, Johan Van Broeck, Aurélia Leroux, Marjorie Gallot, Antoine Bruwier, Hélene Amory, Michel Delmée, Georges Daube

**Affiliations:** Food Science Department, FARAH, Faculty of Veterinary Medicine, University of Liège, Liège, Belgium; Microbiology Unit, Catholic University of Louvain, Brussels, Belgium; Equine Teaching Hospital, Clinical Department of Companion Animals and Equids, FARAH, Faculty of Veterinary Medicine, University of Liège, Liège, Belgium

## Abstract

**Background:**

The equine faecal microbiota is very complex and remains largely unknown, while interspecies interactions have an important contribution to animal health. *Clostridium difficile* has been identified as an important cause of diarrhoea in horses. This study provides further information on the nature of the bacterial communities present in horses developing an episode of diarrhoea. The prevalence of *C. difficile* in hospitalised horses at the time of admission is also reported.

**Results:**

Bacterial diversity of the gut microbiota in diarrhoea is lower than that in non-diarrhoeic horses in terms of species richness (p-value <0.002) and in population evenness (p-value: 0.02). Statistical differences for *Actinobacillus*, *Porphyromonas,* RC9 group*, Roseburia* and *Ruminococcaceae* were revealed. *Fusobacteria* was found in horses with diarrhoea but not in any of the horses with non-diarrheic faeces. In contrast, *Akkermansia* was among the three predominant taxa in all of the horses studied. The overall prevalence of *C. difficile* in the total samples of hospitalised horses at admission was 3.7 % (5/134), with five different PCR-ribotypes identified, including PCR-ribotype 014. Two colonised horses displayed a decreased bacterial species richness compared to the remaining subjects studied, which shared the same *Bacteroides* genus. However, none of the positive animals had diarrhoea at the moment of sampling.

**Conclusions:**

The abundance of some taxa in the faecal microbiota of diarrhoeic horses can be a result of microbiome dysbiosis, and therefore a cause of intestinal disease, or some of these taxa may act as equine enteric pathogens. *Clostridium difficile* colonisation seems to be transient in all of the horses studied, without overgrowth to trigger infection. A large proportion of the sequences were unclassified, showing the complexity of horses’ faecal microbiota.

**Electronic supplementary material:**

The online version of this article (doi:10.1186/s12866-015-0514-5) contains supplementary material, which is available to authorized users.

## Background

Equine gut microbiota is poorly characterised and studies are currently underway to increase understanding of how defined microbial communities are able to interfere with different bacterial aetiologies of diarrhoea in horses. Recently, high-throughput amplicon sequencing analysis has been introduced to investigate the intestinal microbiota of healthy horses and horses with colitis [1–3]. However, the equine faecal microbiome is still largely unknown, as are interspecies interactions and their contribution to animal health [1, 3].

In horses, diarrhoea and colitis have been associated with a number of different pathogenic agents, including *Clostridium difficile, Clostridium perfringens, Salmonella* spp and *Escherichia coli* [4–7]. *Clostridium difficile* is amongst the most important agents of diarrhoea and serious colitis in horses [8]. While both adult horses and foals can suffer *C. difficile* enteric disease, it seems that foals are more likely to be colonised by the bacterium [9]. A diagnosis of *C. difficile* infection (CDI) requires clinical suspicion as well as detection of pre-formed *C. difficile* toxins TcdA and/or TcdB (or transcripts) in non-enriched specimens. Other possible causes of acute colitis must also be ruled out [8]. However, in most cases of diarrhoea, the aetiology remains unclear and the prevalence of *C. difficile* colonisation in hospitalised horses has rarely been addressed [10, 11].

As in humans, the major risk factors for the development of CDI are antibiotic treatment and hospitalisation [8]. However, some cases of infection have been also reported in horses without previous exposure to these risk factors, including in foals at 2 to 5 days of age [12]. Furthermore, it has been reported that up to 7 % of horses carry spores of *C. difficile* without showing any signs of diarrhoea [10, 13].

The first objective of this study was to provide further information on the nature of the bacterial community present in horses developing diarrhoea, including possible alterations in the microbiota profiles as a result of antibiotic treatment, through comparison with faeces from horses without diarrhoea. This study also aimed to examine, by culture of horse faeces at admission to an Equine Clinic, the carriage rate of *C. difficile*. Isolates obtained were characterised in terms of PCR-ribotype, toxigenic activity and antibiotic resistance. Further metagenetic analyses were performed to compare the faecal microbiota of *C. difficile* in colonised and non-colonised horses.

## Results

### Bacterial community present in horses with and without diarrhoea

**Fig. 1:**
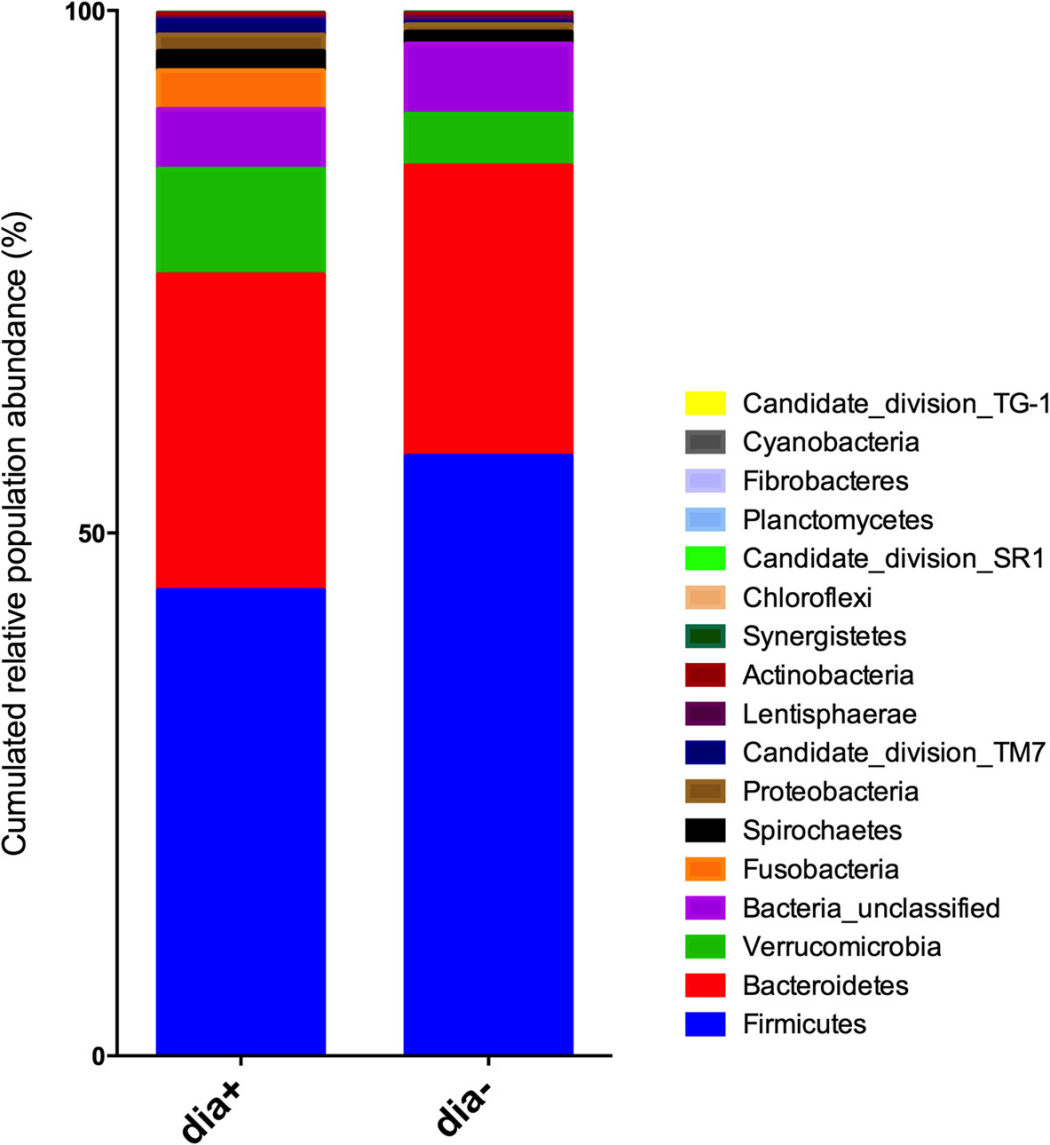
Microbiota faecal composition at phylum level for horses with and without diarrhoea. Bar chart detailing the relative abundance of the 17 core phylotypes common to the two groups of horses (with and without diarrhea)

**Fig. 2:**
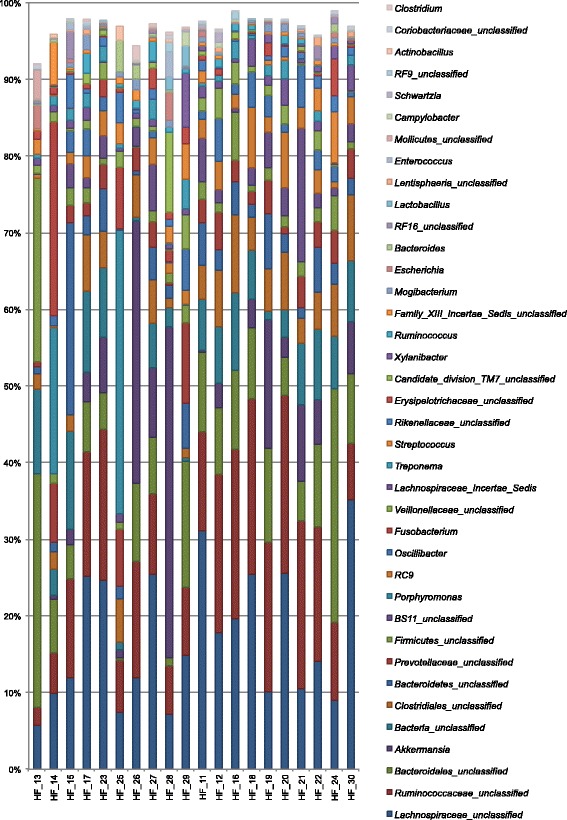
Microbiota faecal composition at genus level (cumulated mean relative abundance >4 %) for horses with and without diarrhoea. Samples 13, 14, 15, 17, 23, 25, 26, 27, 28 and 29: horses with diarrhoea. Samples 11, 12, 16, 18, 19, 20, 21, 22, 24 and 30: horses without diarrhoea. The unclassified populations correspond to defined groups of the genus level for which a taxonomical classification assignation to the genus cannot be attributed. These populations are therefore labelled with the first defined superior hierarchical taxonomic level followed by “_unclassified” to prevent confusion

**Fig. 3:**
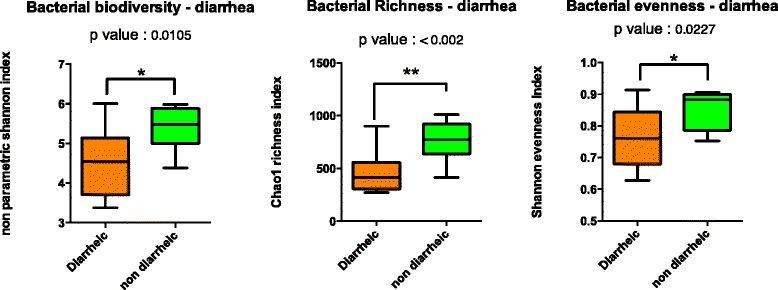
Bacterial biodiversity, bacterial richness and bacterial evenness in *C. difficile* negative horses with and without diarrhoea. Box plot of richness, evenness and diversity values showed that the microbiota structure of each group (diarrhoeic and non diarrhoeic horses) is statistically different from each other. Whiskers represent minimum and maximum value. Bottom and top of the box are the first and the third quartile. The median is shown as a band inside the box

**Fig. 4:**
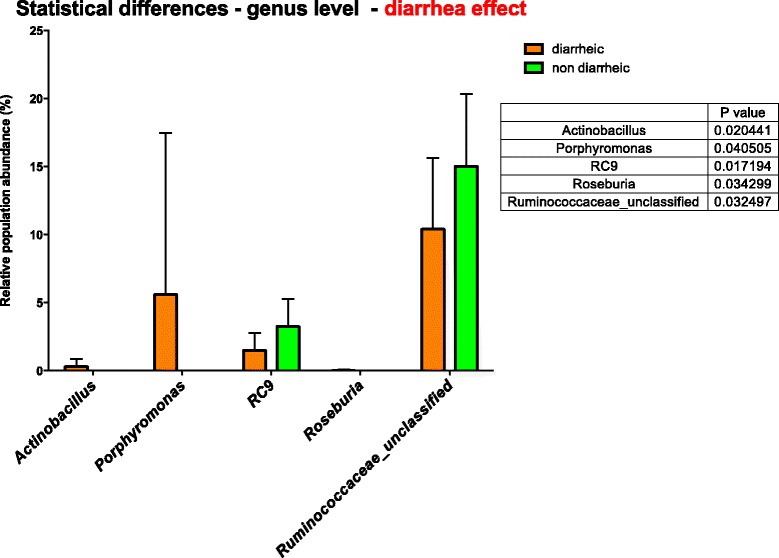
Bacterial genus whose relative abundance was statistically different between the 2 groups. Result of a White test (p value <0.05). Box plot showing mean relative sequence abundance of *Actinobacillus, Porphyromonas*, RC9, *Roseburia* and *Ruminococcaceae_unclassified* in horses with and without diarrhea. The error bar indicates the diversity between samples in terms of proportions of sequences

A group of 10 horses with diarrhoea at the moment of sampling were compared with 10 non-diarrhoeic horses via metagenetic analysis. All of the animals (*n =* 20) tested negative for *C. difficile* by faeces culture (Table 1). Pyrosequencing yielded between 4,000 and 5,000 reads per sample (Additional file 1). The microbiota composition for each horse is presented at phylum level (Fig. 1), genus level (Fig. 2) and species level (Additional file 2). The more abundant bacterial families found for both groups were *Lachnospiraceae* (between 7 % and 39 %)*, Ruminococcaceae* (between 2.7 % and 28.7 %)*, Verrucomicrobiaceae* (between 0 % and 43.1 %), and *Prevotellaceae* (between 0.6 % and 17.5 %). Bacterial diversity of the gut microbiota in diarrhoea was lower than in non-diarrhoeic horses (p-value: 0.0105). This effect was observed both in terms of species richness and in the population evenness (Fig. 3). Principal coordinate analysis (PCoA) of the diarrhoeic and non-diarrhoeic horses did show the sample distribution along the 3 main axes (PC1-PC2-PC3) (Additional file 3). Analysis of Molecular Variance (AMOVA) revealed a significant difference between the variance of both groups taken as a single group and the variance of each group (sum of squares (ss): 0.53 and 11.66 among and within groups respectively; AMOVA test statistic Fs: 1.57; p-value: 0.006). Unifrac weighted analysis further showed that both groups share different population structure (Wscore: 0.88 and p-value: <0.001). Thus, the microbiota structure of each group was showed statistically different from each other. The relative abundance of each genus in both groups were compared in order to identify the populations responsible for this difference (White *t*-test), leading to statistical differences for *Actinobacillus*, *Porphyromonas,* RC9 gut group*, Roseburia* and a taxonomically undefined population belonging to the *Ruminococcaceae* (*Ruminococcaceae* unclassified) (Fig. 4).

**Table 1:** Detailed information on twenty *C. difficile* negative horses studied via high-throughput amplicons sequencing analysis with and without diarrhoea

Clinical history of horses							
Date of sampling	Animal number	Age (years)	Diagnostic	Diarrhea	Hospital stay (days)	Antibiotic treatment (days) ^1^	NSAIDs treatment
9/10/13	13	12	Colic	+	5	Pen-Gen (1)^2^	F-M
21/10/13	14	3	Diarrhoea	+	3	-	-
28/10/13	15	21	Diarrhoea and colic	+	2	SXT (2)	F-M
5/11/13	17	13	Equine atypical myopathy	+	1	-	-
18/11/13	23	9	Diarrhoea and colic	+	1	-	Dipyrone
18/11/13	25	2	Haemorrhagic enterocolitis	+	1	-	-
			Hyperlipemia				
22/11/13	26	5	Diarrhoea and weight loss	+	1	Xnl (1)	F-M
							Dipyrone
26/11/13	27	6	Colic	+	2	-	F-M
26/11/13	28	11	Colic	+	4	Pen-Gen-LZ (1)^2^	F-M
							Dipyrone
							Firocoxib
19/12/13	29	2	Diarrhoea	+	2	-	-
8/10/13	11	11	Oesophageal obstruction	-	1	Pen-Gen-LZ (1)^2^	F-M
9/10/13	12	8	Horse fall	-	10	Xnl (7)^2^	Dipyrone
28/10/13	16	21	Wound	-	3	SXT (4)^2^	Dipyrone
7/11/13	18	8	Equine atypical myopathy	-	1	-	-
8/11/13	19	6	Colic	-	1	-	-
12/11/13	20	6	Colic	-	1	-	F-M
13/11/13	21	5	Equine atypical myopathy	-	1	-	-
18/11/13	22	3	Osteochondritis dissecans screening	-	1	-	-
18/11/13	24	9	Colic	-	2	Pen-Gen (2)^2^	F-M
19/12/13	30	2	Arthroscopy	-	3	-	-

*NSAIDs* nonsteroidal anti-inflamatory dugs

*Pen* penicillin, *Gen* gentamicin, *Xnl* ceftiofur, *SXT* trimethoprim/sulfamethoxazole, *LZ* metronidazole

*F-M* Flunixin meglumine

^1^Time before antibiotic administration in days ^2^Antibiotic treatment in progress at the time of sampling

### *Clostridium difficile* prevalence in horses at admission and strain characterisation

During the three-month study period, the total number of horses admitted at the clinic for either emergencies or consultation was 302, with 141 hospitalisations. A total of 136 samples were collected from the 134 hospitalised horses enrolled in the study. Two horses were sampled on two different times because they suffered a diarrhoeal episode during hospitalisation. However, these horses did not test positive for *C. difficile* on any of the sample days. The overall prevalence of *C. difficile* in the faecal microbiota of hospitalised horses at admission was 3.7 % (5/134). All horses testing positive were adult animals aged between four and 16 years old.

Altogether, 52 of the total of 134 horses studied (38.8 %) presented gastrointestinal problems at admission, but *C. difficile* was isolated from only three of these animals (two with colic and one with proximal enteritis). In these three horses a nasogastric tube had been passed before the faecal collection. However, none of them had previously received an antibiotic treatment. Nineteen horses (14.2 %) had diarrhoea at admission but all tested negative for the bacterium (Additional File 4).

The remaining 82 horses sampled (61.2 %) were not affected by gastrointestinal disorders. In this group, *C. difficile* was detected in two horses. The clinical diagnoses in these two infected horses were multiple wounds and wound with tendon injury, respectively. Both had received a similar antibiotic treatment (gentamicin and penicillin with or without ceftiofur) and non-steroidal anti-inflammatory drugs (dypirone). Age (9 years) and the gender (female) of the two horses were also the same (Table 2). In terms of antimicrobial therapy, 54 out of 134 horses studied (40.3 %) had previously received antimicrobial therapy (prior to the study period), but most (*n =* 52) tested negative for *C. difficile.* Among them, the most common drug used was ceftiofur, which was administered to 16 (11.9 %) horses.

**Table 2:** Detailed information on *C. difficile* positive horses, including molecular characterization and antibiotic resistance of the isolates

*C. difficile* positive horses								Isolates characterisation										
Date of sampling	Animal identification	Age (years)	Diagnostic	Diarrhoea	Hospital stay (days)	Antibiotic treatment (days)^e^	AINS treatment	PCR-ribotype	CE	*tcdA tcdB*	*cdtA cdtB*	*tcdC* ^a^ *gyrA* ^b^ MUT	PEN	CC	TE	VA MXF RA	LZE	XNL
15/10/2013	01	9	Wound and tendon injury	-	1	Pen-Gen-Xnl (1)^f^	Dipyrone	UCL237	+	+	-	-	I	R	I	S	S	R
05/11/2013	03	16	Colic^c^	-	1	-	Flunixin meglumine	UCL49	+	+	-	-	I	R	S	S	S	R
12/11/2013	04	4	Colic^d^	-	2	-	-	014	+	+	-	-	I	S	S	S	S	R
03/12/2013	09	9	Multiple wounds	-	4	Pen-Gen (5)^f^	Dipyrone	UCL36	-	-	-	-	I	R	S	S	R	R
09/12/2013	10	4	Proximal enteritis	-	4	-	Firocoxib Meloxidyl Dipyrone	UCL23f	+	+	-	-	I	R	S	S	S	R

*MUT* mutation

*CE* cytotoxicity assay using confluent monolayer MRC-5 cells

*Gen* gentamicin, *Pen* penicillin, *CC* clindamycin, *TE* tetracycline, *VA* vancomycin, *MXF* moxifloxacin, *RA* rifampicin, *LZ* metronidazole, *E* erythromycin, *XNL* ceftiofur

*I*intermediate antimicrobial resistance

^a^Presence of deletions in the regulator gene *tcdC* (118 bp-39 bp-17 bp)

^b^Presence of mutation in the *gyrA* gene associated with moxifloxacin resistance

^c^Colic secondary to a gaseous distension of the caecum and a retraction of the colon

^d^Colic secondary to a pelvic flexure impaction (suspicion of a digestive tract verminosis causing weight loss)

^e^Time before antibiotic administration in days ^f^Antibiotic treatment in progress at the time of sampling

Four of the equine isolates were positive for *tcdA, tcdB* and binary toxin CDT genes while only one was non-toxinogenic. The presence of TcdB was confirmed by cytotoxicity assay using confluent monolayer MRC-5 cells. None of the isolates presented an 18, 39-base pair deletion or a deletion at 117 of the *tcdC* gene. Five different PCR-ribotypes were detected. Only one strain had a ribotype profile associated with the reference Cardiff collection number (014). The remaining isolates were identified as UCL237, UCL49, UCL23f and UCL36 (non-toxigenic PCR-ribotype). PCR-ribotypes UCL49, 014 and UCL23f were isolated from three animals with gastrointestinal problems while PCR-ribotypes UCL237 and UCL36 were recovered from the two horses with wounds (Table 2).

Only the non-toxigenic strain PCR-ribotype UCL36 showed resistance to metronidazole (minimum inhibitory concentration (MIC) = 40 μg/ml, average of two essays) and erythromycin. For clindamycin, only one isolate (PCR-ribotype 014) was susceptible, while all others were fully resistant. Intermediate resistance for penicillin was observed in all of the isolates tested. The isolate PCR-ribotype UCL237 also exhibited intermediate resistance to tetracycline, while all the rest were susceptible. There was no vancomycin, moxifloxacin or rifampicin resistance detected, but all the strains were resistant to ceftiofur (Table 2).

### Microbiota composition for *C. difficile* positive and negative horses

**Fig. 5:**
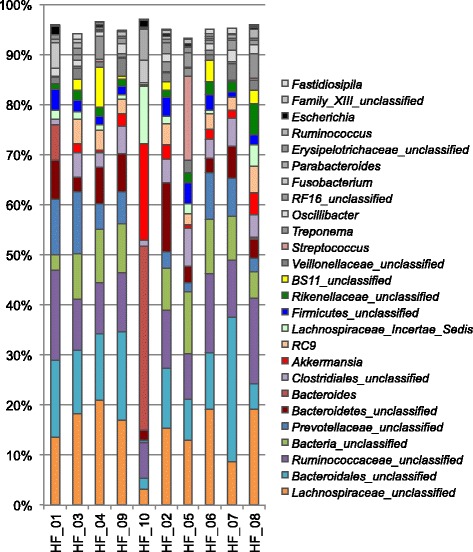
Microbiota faecal composition at genus level (cumulated mean relative abundance >4 %) of *C. difficile* culture-positive and -negative horses. Samples HF_01, 03, 04, 09 and 10: *C. difficile* positive horses detected by faeces culture. Samples HF_02, 05, 06, 07 and 08: *C. difficile* negative horses detected by faeces culture

Stool samples from all horses testing positive for *C. difficile* (*n =* 5) were studied in order to obtain further information about the microbiota composition of the colonised subjects. As during the entire study period (3 months) only five animals were positive for the bacterium, we could only use five *C. difficile*-negative horses as control group, but with similar clinical history (Additional file 5). In both *C. difficile* colonised and non-colonised horses, the dominant taxa were *Lachnospiraceae* (ranging from 3.2 to 20.8 %), *Bacteroidales* (ranging from 5 to 29.0 %) and *Ruminococcaceae* (ranging from 7 to 17.9 %). In the group of *C. difficile* positive horses, only one animal (10) presented a predominance of *Bacteroides* (36.8 %) and *Akkermansia* (19.5 %). The same *Bacteroides* genus was found in another *C. difficile* positive sample (01) at a level of 7.2 % (Fig. 1). Only 45 distinct OTUs (with a mean abundance greater than 4 %) were identified, representing 25-40 % of the relative abundance of the microbial taxa in the faecal samples (Fig. 5 and Additional file 6).

However, bacterial biodiversity, bacterial richness and bacterial evenness were not statistically different between *C. difficile* colonised and non-colonised horses (p-value > 0.05). Ordination analysis with PCoA supported the grouping of most of the individuals into one group (Additional file 7), which confirmed by non significant results from AMOVA (sum of squares (ss) 0.41 and 3.18 among and within groups respectively; test statistic for AMOVA (Fs) 1.03; p-value: 0.389) and Unifrac analysis of sample clustering (Wscore: 0.97 and p-value: 0.92). Indeed, White test abundance population comparison between groups performed at the different taxonomical levels identified two taxa (RF16 group and *Clostridiales*) for which the relative abundance was statistically different (Additional file 8).

### Microbiota composition relation with gastro-intestinal disorder diagnostics

We grouped the microbiota profiling data from the 20 samples from diarrhoeic and non diarrhoeic samples with the 10 *C. difficile* positive and negative samples together and assigned them to diagnosis categories: Colic (*n =* 12), Enteritis (*n =* 1), Enterocolitis (*n =* 1), diarrhoea (*n =* 3) and others (13). We used this clustering in order to identify genus whose abundance could be related to one particular category. Statistical abundance comparison with ANOVA2 between colic, diarrhoea and other categories highlight a higher abundance of *Escherichia* and *Streptococcus* genera in colic group compared to the others and higher abundance of *Akkermansia*, *Fusobacterium*, *Porphyromonas* and *Xylanibacter* genera in the Diarrhoea group (Additional file 9). Enteritis and Enterocolitis group, having only one sample could not be included in statistical abundance comparison but each sample was dominated by 2 defined genus: *Bacteroides* and *Parabacteroides* in the Enteritis sample and *Porphyromonas* and *Fusobacterium* Enterocolitis sample (Additional file 10).

## Discussion

High-throughput amplicon sequencing analysis is one of the methods of choice in the study of complex gut microbiota ecosystems [14]. However, most of the studies reported bacteria populations at the phylum and class level while the genus and species level were explored only in a few recent studies [15, 16]. Higher taxonomic resolutions (genus or species level) may reveal more differences in population structure than phylum or class level [17] and provide the degree of precision necessary for clinical diagnosis [18]. As previously demonstrated, selecting the accurate region of 16S ribosomal DNA (rDNA) gene to sequence is essential in determining the utility of microbial genomics for species-level assignments [19]. In the present study we report genus and species labelling based on V1-V3 region.

To determine whether the presence of diarrhoea was related to changes in the composition of the faecal microbiota of horses, a strict screening process was carried out among all the samples obtained over three months, in order that the two groups (with and without diarrhoea) were as similar as possible and therefore comparable. As observed in a previous study investigating the microbiota in the equine large intestine via 16S ribosomal DNA sequencing [1], a great part of the sequences (60 %) were not identical (less than 1 % mismatch) to sequence entries present in SILVA database (v1.15). Even among the sequences identical to known entries, the species name was seldom taxonomically defined. These findings underline the lack of knowledge regarding a good part of the horse gut microbiota stressing the need for further research on fundamental microbiology either on taxonomic as well on the functional level.

Interestingly, *Akkermansia* was found in 90 % of horses studied, with a relative abundance ranging between 0.03 % and 43.1 %. This bacterium is an appealing candidate to become a human probiotic, selected based on established mechanisms of preventative treatment of obesity and diabetes [20–22]. Only one previous study [23] describes the genus *Akkermansia* in the equine intestinal microbiota In this study we reported the presence of *Akkermansia muciniphila* and *Akkermansia EU779370* in the faecal microbiota of horses with and without diarrhoea*. Akkermansia EU779370* population identified in this study is 100 % identical to the *Akkermansia EU779370* GenBank entry (for the sequenced V1-V3 section). It represents a potential new *Akkermansia* species as it shares only 90 % of nucleotide identity with *Akkermansia muciniphila.* The relevance of this finding deserves further investigation.

Overall, the composition of the microbiota of all horses studied was dominated by the same taxa as previously described [4]. However, the differences observed in the cumulative mean relative abundance among individuals in these dominant bacterial groups may be linked to recent dietary history [17] or to antimicrobial therapy [23]. Diarrhoea has been associated with changes in the faecal microbiota composition of humans but alterations in the equine gut microbiota has been rarely addressed [24]. In the present study, *Actinobacillus* (0.3 % mean abundance in diarrhoeic group versus 0.004 in non-diarrhoeic group) and *Porphyromonas* taxa (5.6 % mean abundance in diarrheic group versus 0.002 in non-diarrhoeic group) were detected more abundantly in horses with diarrhoea. These results contrast with a previous study of commensal bacteria in acute diarrhoea in children, where *Porphyromonas* species were in the lowest proportions during acute diarrhoea compared with levels during periods of normal gastrointestinal health [25]. *Fusobacteria* were found in horses with diarrhoea but not in any of the horses with normal faeces. In a previous study, *Fusobacterium* spp. was also found in higher percentages in horses with colitis, which could be a consequence of overgrowth due to bacterial dysbiosis or an aetiological agent of disease [4]. In human beings, *Fusobacteria* have been associated with colorectal carcinomas and adenomas [26]. However, there are no previous studies describing this bacterium as an equine enteropathogen [4].

After clustering of the different feces samples into more defined diagnosis categories, genus *Escherichia* and *Streptococcus* were more abundant in horses with diarrhoea without any other symptoms. A more refined analysis revealed that the species involved were *Escherichia coli* and *Streptococcus equinus*. If the first is long known to be associated with diarrhoea, the second is the most common *Streptococcus* found in horse feces. In the colic group, *Fusobacterium* and *Porphyromonas* genera were found in higher amount compared to other groups but were composed of yet unknown species. As mentioned above, little is known regarding the involvement of these bacteria in gastro-intestinal disorders. Finally, two horses suffered from enteritis and enterocolitis and their 16S profiling revealed a domination (above 30 % of the sample sequences) of genus *Bacteroides* in the enteritis case and genus *Porphyromonas* in the enterocolitis case. The *Bacteroides* population was mainly represented by *Bacteroides heparinolyticus*. This species, originally characterised as an agent of periodontitis in human [27], is phylogenetically related to *Bacteroides fragilis* which is well-known enterotoxinogenic bacteria involved in human infections [28]. However, there is still no evidence of *B. heparinolyticus* involvement in gut disorder in horse or in human and it is thus unclear whether its abundance is related to the symptomatology.

We are aware that the limited size of the analysed cohort reduces the strength and the scope of our results. Larger cohort studies will be needed to improve our knowledge on diarrhoea impact on horse microbiota.

In the present study, the carriage of *C. difficile* at the time of admission was examined. The overall prevalence found was 3.7 % (5/134). None of the positive animals had diarrhoea at the moment of sampling which may suggest that *C. difficile* colonisation in these horses was transient in most cases. In the literature, there is only one previous study that investigated the presence of *C. difficile* in horses at admission to a veterinary teaching hospital [11]. Our results correlate with the findings of this study, which reported a prevalence of 4.8 % (4/82). In another previous study conducted at the same Belgian Equine Clinic, we observed a *C. difficile* colonisation rate of 13.7 % (10/73). However, in that study, animals were not only sampled at the time of admission but also tracked during their hospitalisation, which could explain the higher prevalence found [10].

From the five *C. difficile* positive horses found, three of them presented gastrointestinal problems (colic and proximal enteritis) with a nasogastric tube passed before sampling. Nasogastric tube placement has been previously identified as a risk factor for *C. difficile* infection [11]. Two other horses without enteric perturbations were also colonised by *C. difficile.* Both had suffered wounds and were treated with antibiotics. Previous studies reported intestinal flora perturbations and antibiotic exposure as the most significant risk factors for *C. difficile* proliferation in horses [5].

There were five different PCR-ribotypes detected among the five *C. difficile* positive animals, which suggests that a wide variety of *C. difficile* strains circulate in horses, as previously reported [11, 29], including PCR-ribotype 014. All the isolates were resistant to ceftiofur and four out of five were also resistant to clindamycin, which agrees with the findings of previous studies [10, 30]. Only one isolate was resistant to metronidazole and erythromycin. Surprisingly, this strain (PCR-ribotype UCL36) was the only non-toxigenic isolate. A high degree of resistance to antimicrobials (including erythromycin and clindamycin) in non-toxigenic strains has been reported previously [30] but the role in disease development or prevention is still unknown [31].

We were unable to identify *C. difficile* by pyrosequencing analysis in the stool samples with positive cultures*.* In humans, it is considered that feces harbour up to 10^12^ bacteria per gram [32]. Thus, our sampling of thousands of sequences limits our detection ability to populations above 10^9^ bacteria per gram. The horses testing positive by culture did not have any clinical signs of *C. difficile* disease and the isolate was obtained only after three days of enrichment. While the results of high-throughput amplicon sequencing analysis are limited by the small number of animals positive for *C. difficile*, and by the fact that none of the animals suffered CDI, the findings for each colonised horse should not be dismissed as they provide a first insight, albeit limited, about the impact of *C. difficile* colonisation in the horses’ gut microbiota, which merits further investigation.

## Conclusions

Metagenomic analysis is a promising tool to identify correlations between changes in the gut microbiota and intestinal diseases. The abundance of *Actinobacillus, Porphyromonas* and *Fusobacteria* in the faecal microbiota of diarrhoeic horses deserves special attention, as it can be a result of microbiome dysbiosis, and therefore a cause of intestinal disease, or in the case of *Fusobacteria*, may act as equine enteric pathogen. Furthermore, the high proportion of *Akkermansia* in all of the horses studied and its role in the intestine merits further investigation. *Clostridium difficile* colonisation seems to be transient in all of the horses studied without overgrowth to trigger infection. For a great variety of bacterial species the currently available systems are not able to confidently assign taxonomy, which shows how complex and still unknown the equine microbiome is.

## Methods

### Inclusion criteria and sampling

Samples were collected over a three month period (October to December 2013) at the Equine Clinic, Department of Companion Animals and Equids, Faculty of Veterinary Medicine, University of Liege.

All hospitalised horses during this period with a clinic stay of at least one day were eligible. Subjects were all selected without regard to their diagnosis or the possible duration of hospitalisation. The exclusion criteria were horses exhibiting dysphoric mood, intolerable stress, or any other disease for which sampling by rectal manipulations was not recommended. Samples were collected between day one and day two following admission. In addition, all horses developing an episode of diarrhoea during their hospital stay were sampled for a second time. Horses were documented for data relating to clinical history, diagnostic findings and treatment received, including the prescription of antimicrobial agents. Faecal sampling was performed directly via rectal. Samples obtained were scored as normal faeces, diarrhoea or bloody diarrhoea faeces. All samples were processed on the same day immediately after transport (at room temperature) to the laboratory (approximately 15 min after sampling). In cases of emergency admission (i.e., during the night or on a non*-*working day)*,* two samples per horse were collected. The first sample was collected in an individual identified sterile 50 ml tube for further culture to detect *C. difficile*; the second was collected using the Stool DNA stabiliser (PSP^R^ Spin Stool DNA Plus Kit 00310, Invitek) and stored at 4 °C in the hospital for a maximum of three days before processing. After culture of faeces, all samples were frozen immediately at −80 °C before DNA extraction.

### 16S rDNA pyrosequencing and data analysis

Among all the faecal samples collected, clinical history of each subject was investigated in order to select two homogenous groups of horses (with and without diarrhoea) with the same number of individuals in each group. Both groups were matched for age, pathologies, previous hospital stay and medical treatment. A total of 20 faecal samples (ten with diarrhoea and ten without diarrhoea) were further studied. A second selection was done on the pool of 140 horses to gather 2 homogenous groups of horses, either or not positive for *C. difficile* by classical microbiology (*n =* 5), which were matched for age, pathologies, previous hospital stay and medical treatment. Finally, the 30 samples were grouped together in a third analysis and clustered into defined diagnostic category based upon diagnostic (Table 1; Table 2; Additional file 5). The resulting diagnostic categories are: colic—horses suffering from abdominal pain (*n =* 12); diarrhoea—horses with 3 or more loose or liquid stools per day, without other symptom (*n =* 3); enteritis—horse with ileon inflammation (*n =* 1); enterocolitis—horse with ileon and colon inflammation (*n =* 1) and others—horses with non Gastro-intestinal disorders (*n =* 13).

Total DNA was extracted from the stool samples with the PSP^R^ Spin Stool DNA Plus Kit 00310 (Invitek), following the manufacturer’s recommendations. The DNA was eluted into DNase/RNase-free water and its concentration and purity were evaluated by absorbance measurement using the NanoDrop ND-1000 spectrophotometer (NanoDrop ND-1000, Isogen). PCR-amplification of the V1-V3 region of the 16S rDNA was performed as previously described [33]. Primers E9-29 and E514-530 [33–35] were selected for their theoretical ability to generate the lowest possible amplification capability bias among the various bacterial phyla [36]. The oligonucleotide design included 454 Life Sciences’ A or B sequencing titanium adapters (Roche Diagnostics) and multiplex identifiers (MIDs) fused to the 5′ end of each primer. The master mix composition consisted of 5 units of FastStart high fidelity polymerase (Roche Diagnostics), 1x enzyme reaction buffer, 200 μM dNTPs (Eurogentec), 0.2 μM of each primer and 100 ng of genomic DNA in a volume of 100 μl. The amplification was carried out in a gradient thermocycler (Eppendorf) as follows: denaturation at 94 °C for 15 min followed by 25 cycles of 94 °C for 40 s, 56 °C for 40 s; 72 °C for 1 min; and a final elongation step at 72 °C for 7 min. PCR products were run on 1 % agarose gel electrophoresis and purified using the SV PCR purification kit (Promega Benelux). Picogreen dsDNA quantitation assay (Isogen) was performed in order to assess the quality and quantity of the products. All libraries were run in the same titanium pyrosequencing reaction using Roche multiplex identifiers, and amplicons were sequenced using the Roche GS-Junior Genome Sequencer instrument (Roche).

Sequence reads were processed using MOTHUR software package v1.32 [37] and denoised using the Pyronoise algorithm [38]. Trimming criteria of the reads was applied as follows: read lengths no shorter than 425 bp, an exact match to the barcode, and 1 mismatch allowed to the proximal primer [33]. Sequences were checked for the presence of chimeric amplifications using the UCHIME algorithm [39].

The read sets obtained were compared with a reference data set of aligned sequences of the corresponding region derived from the SILVA database (v1.15) of full-length rDNA sequences [40] implemented in MOTHUR [41]. The final reads were clustered into operational taxonomic units (OTUs) using the nearest MOTHUR neighbour algorithm with a 0.03 distance unit cut-off. Taxonomic identity was attributed to each OTU by comparison with the SILVA v1.15 database [37] (80 % homogeneity cut-off) [33]. When taxonomic identification fell below the 80 % treshold, the taxonomic level was labelled with the first defined level from higher level followed by the term “_unclassified”.

All unique sequences for each OTU were further compared with the SILVA data set version v1.15 using the BLASTN algorithm [42, 43], as MOTHUR is not suitable to taxonomic assignment beyond the genus level. For each OTU, a consensus detailed taxonomic identification was given based upon the identity (less than 1 % of mismatch with the aligned sequence) and the metadata associated with the most frequent hits leading to 3 kind of labelling : (i) the population is identical to a taxonomically defined species and is labelled “genus_species”; (ii) the population is identical to a reference sequence belonging to a still undefined species and is labelled “genus_NCBI Accession Number”; (iii) the sequence is not identical to any known sequence and is arbitrarily labelled with its OTU number [33].

Subsample datasets were obtained and used to evaluate ecological indicators (the richness and microbial diversity of the samples) using MOTHUR. Population structure and community membership were assessed with MOTHUR using distance matrices based on the Jaccard index (a measure of community membership; which considers the number of shared OTUs but not their abundance) and the Yue and Clayton measure of dissimilarity (a measure of community structure which considers shared OTUs and their relative abundances) [44]. Richness estimation (Chao1 estimator) [45], microbial biodiversity (non-parametric (NP) Shannon diversity index) [46], and the population evenness (Shannon evenness) [47] were calculated using MOTHUR. Chao 1 estimator was used to estimate the richness of the detected species (OTUs) in a sample [33].

### Ordination and statistical analysis and biosample accession numbers

Ordination analysis were performed with Vegan package in R [48].

Principal coordinate analysis (PCoA) was applied to visualise the biodiversity between the two groups [49]. Statistical analysis regarding community structure and composition were performed with AMOVA and UNIFRAC implemented in MOTHUR v1.32. Analysis of molecular variance (AMOVA) was used for estimating population differentiation [50]. Unifrac unweighted analysis, which accounts for the relative abundance of each of the taxa within communities, was used to evaluate differences in population structure between pairs of sample categories [51]. The differences were considered significant for a p-value of less than 0.05; all the results given are the means ± the standard deviations of the results between the samples of each category [33]. Statistical differences between bacterial biodiversity, richness and evenness were assessed using two-sided unpaired *t*-test using PRISM 6 (Graphpad Software). In order to highlight statistical differences in the bacterial population abundance between categories, a White tests (paired comparisons) and ANOVA with Tukey post-hoc test were performed using STAMP software [52]. All the biosample sequences have been deposited at the National Center for Biotechnology Information (NCBI) [53] and are available under de Bioproject ID PRJNA279335.

### *Clostridium difficile* culture, identification and characterisation

For isolation of *C. difficile*, one gram of faeces was inoculated into 9 ml of CCFT (cycloserine cefoxitin fructose taurocholate) broth as previously described [54] and incubated anaerobically for 72 h at 37 °C. A 10 μl aliquot of the enriched broth was spread on CCFT plates and incubated anaerobically at 37 °C for three days. One presumptive colony per plate was subcultured onto blood agar 5 % Sheep Blood (Biorad) and checked using a *C. difficile* latex agglutination rapid test Kit DR 1107A (Oxoid). Identification, toxin gene profile, deletions in the regulator gene *tcdC,* and *gyrA* mutation (gene associated with moxifloxacin resistance) were determined using the Genotype Cdiff system (Hain Lifescience) according to the manufacturer’s instructions. The supernatant from each pure culture was tested for cytotoxicity assay (TcdB) using confluent monolayer MRC-5 cells, as previously described [54]. A PCR ribotyping method based on capillary gel was performed using the primers recommended by Bidet et al. [55]. The isolates with a PCR-ribotype profile from which reference strains were available in our laboratory were designated with the relevant Cardiff international number. Otherwise, isolates were identified with an internal nomenclature beginning with UCL.

### Antibiotic resistance

*Clostridium difficile* isolates (*n =* 5) were tested for susceptibilities to a panel of nine antimicrobial agents by disc diffusion (*n =* 7) and E-test (*n =* 2). The antimicrobials studied were chosen because they have been associated with *C. difficile* infection or its treatment, or because they were widely used in the equine teaching hospital used in the study.

Resistance to rifampin (25 μg), erythromycin (15 μg), oxitetracycline (30 μg), vancomycin (30 μg), penicillin (10 μg), clindamycin (2 μg), and ceftiofur (30 μg) (Becton-Dickinson) was tested by disc diffusion assay on Brucella Blood Agar with hemin and vitamin K1 (Becton-Dickinson) according to the French Society of Microbiology [56] protocols. Zone diameters were read after 24 h of anaerobic incubation at 37 °C and interpreted as previously described [10].

Susceptibility to metronidazole and moxifloxacin was determined using the Etest method (Lucron ELITechGroup) on Schaedler with Vit K1 and 5 % sheep blood (Becton-Dickinson) according to the manufacturer’s instructions. Plates were incubated anaerobically at 37 °C for 48 h. The susceptibility and resistance breakpoints for metronidazole (s ≤8 μg/ml; r ≥32 μg/ml) and moxifloxacin (s ≤ 2 μg/ml; r ≥8 μg/ml) used for interpretation were those recommended by the Clinical and Laboratory Standard Institute [57]. *Bacteroides fragilis* ATCC 25285 was tested as a quality control.

### Metagenetic analysis

Rectal faecal samples of all animals positive for *C. difficile* (*n =* 5) were analysed by 16S rDNA pyrosequencing (as described above) in order to recover further information about the microbiota composition of horses colonised by the bacterium. A further group of non-colonised horses (*n =* 5) was used as a control. Control subjects were selected on basis of their similarity to colonised horses, including same clinical history (pathologies and antimicrobial treatment), age and previous hospital stay, to obtain two groups as similar as possible.

### Ethics

This study required no experimental research on animals, only the use of collected feces. Therefore, this study did not require approval from Animal Ethics Committee following Belgian (royal decree M.B.10.07.2013) and European legislation (2010/63/UE).

## Additional files

Additional file 1:
**Quality analysis of the metagenetic libraries created for the horse faecal samples analysed.** (DOCX 35 kb) Additional file 2:
**Microbiota faecal composition at species level (cumulative mean relative abundance >4 %) of horses with and without diarrhoea.** Samples 13, 14, 15, 17, 23, 25, 26, 27, 28 and 29: horses with diarrhoea. Samples 11, 12, 16, 18, 19, 20, 21, 22, 24 and 30: horses without diarrhoea. (DOCX 146 kb) Additional file 3:
**Principal coordinate analysis of the diarrhoeic and non-diarrhoeic horses.** The 3 graphs show the sample distribution along the 3 main axes (PC1-PC2-PC3). (DOCX 83 kb) Additional file 4:
**Clinical history comparison between** ***C. difficile*** **colonised and non-colonised horses.** ^a^Antimicrobial treatment of two or more antibiotics. ^b^Including single or combined antimicrobial therapy (DOCX 63 kb) Additional file 5:
**Detailed information on five** ***C. difficile*** **negative horses studied via high-throughput amplicon sequencing analysis and compared with** ***C. difficile*** **colonised horses.** NSAIDs: nonsteroidal anti-inflammatory drugs. Pen: penicillin; Gen: gentamicin; SXT: trimethoprim/sulfamethoxazole (DOCX 46 kb) Additional file 6:
**Microbiota faecal composition at species level (cumulative mean relative abundance >4 %) of** ***C. difficile*** **culture-positive and -negative horses.** Samples 01, 03, 04, 09 and 10: *C. difficile* positive horses detected by faeces culture. Samples 02, 05, 06, 07 and 08: *C. difficile* negative horses detected by faeces culture. (DOCX 161 kb) Additional file 7:
**Principal coordinate analysis of** ***C. difficile*** **positive and negative horses.** The 3 graphs show the sample distribution along the 3 main axes (PC1-PC2-PC3). (DOCX 73 kb) Additional file 8:
**Bacterial genus whose relative abundance was statistically different between** ***C. difficile*** **positive and negative horses.** Result of a White test (p value <0.05) pairwise comparison. (DOCX 95 kb) Additional file 9:
**Bacterial genus whose relative abundance was statistically different between Colic, diarrhoea and Others diagnosis groups.** The graph illustrates the mean relative abundance for the selected genera. Groups were compared by ANOVA 2 analysis with post-hoc Tukey-kramer multiple comparison. The inside table shows the pairwise difference (p-value < 0.05) represented by different letters. (DOCX 159 kb) Additional file 10:
**Abundance of the dominant genera found in the enteritis and enterocolitis samples.** The graph shows the relative abundance of the 2 dominant genera found in enteritis and enterocolitis samples as well as their mean relative abundance in the other diagnosis categories. (DOCX 132 kb)
