# Supplementary material for: Faecal microbiota characterisation of horses using 16 rdna barcoded pyrosequencing, and carriage rate of clostridium difficile at hospital admission
Source: BMC Microbiol. 2015 Sep 16;15:181. doi: 10.1186/s12866-015-0514-5 (PMC4573688; doi:10.1186/s12866-015-0514-5)
Supplement: Additional file 1: — Quality analysis of the metagenetic libraries created for the horse faecal samples analysed. (DOCX 35 kb) [file 12866_2015_514_MOESM1_ESM.docx]

| Item | Total number (or %) | Mean read length, nucleotide |
| --- | --- | --- |
| Raw reads | 137,319 | 510 |
| Postdenoising/filtering | 118,429 | 452 |
| Loss in denoising (%) | 13.8 |  |
| Postchimeric detection | 104,608 | 450 |
| Chimeric reads (%) | 11.7 |  |
| OTU 0.03^a^ | 8,086 |  |
