# Supplementary material for: Faecal microbiota characterisation of horses using 16 rdna barcoded pyrosequencing, and carriage rate of clostridium difficile at hospital admission
Source: BMC Microbiol. 2015 Sep 16;15:181. doi: 10.1186/s12866-015-0514-5 (PMC4573688; doi:10.1186/s12866-015-0514-5)
Supplement: Additional file 4: — Clinical history comparison between C. difficile colonised and non-colonised horses. aAntimicrobial treatment of two or more antibiotics. bIncluding single or combined antimicrobial therapy (DOCX 63 kb) [file 12866_2015_514_MOESM4_ESM.docx]

|  | *C. difficile* negative horses (%) | *C. difficile* positive horses (%) |
| --- | --- | --- |
| Overall totals (%) | 129 of 134 (96.3%) | 5 of 134 (3.7%) |
| Mean age in years | 10.2 | 8.4 |
| Sort by size |  |  |
| Heavy horses (>650kg) | 16 (12.4) | 0 (0) |
| Light horses (<650kg) | 94 (72.8) | 4 (80) |
| Ponies/donkeys | 19 (14.7) | 1 (20) |
| Sort by gender |  |  |
| Mare | 62 (48.9) | 3 (60) |
| Stallion | 24 (18.6) | 0 (0) |
| Gelding | 43 (33.3) | 2 (40) |
| Horses with gastrointestinal disorders 52/134 (38.8) | 49 (94.2) | 3 (5.8) |
| Colic | 30 (61.2) | 2 (66.6) |
| Diarrhoea | 18 (36.7) | 0 (0) |
| Colic and diarrhoea | 1 (2.0) | 0 (0) |
| Others | 0 (0) | 1 (33.3) |
| Horses without gastrointestinal disorders 82/134 (61.2) | 79 (96.3) | 3 (3.7) |
| Orthopaedics/bone fracture | 23 (29.1) | 0 (0) |
| Wounds | 14 (17.7) | 2 (66.6) |
| Muscular dystrophy | 8 (10.1) | 0 (0) |
| Ophthalmology | 8 (10.1) | 0 (0) |
| Weight loss | 3 (3.8) | 0 (0) |
| Others | 23 (29.1) | 1 (33.3) |
| Horses with an antibiotic treatment 54/134 (40.3) | 52 (96.3) | 2 (3.7) |
| Penicillin | 4 (7.7) | 0 (0) |
| Penicillin-Gentamicin^a^ | 9 (17.3) | 1 (50) |
| Penicillin-Gentamicin-Metronidazole^a^ | 3 (5.8) | 0 (0) |
| Ceftiofur | 16 (30.8) | 0 (0) |
| Trimethoprim/sulfamethoxazole | 6 (11.5) | 0 (0) |
| Others^v^ | 14 (27) | 1 (50) |
| Placement of a nasogastric tube 43/134 (32.1) | 40 (93) | 3 (7) |
