# Supplementary material for: Faecal microbiota characterisation of horses using 16 rdna barcoded pyrosequencing, and carriage rate of clostridium difficile at hospital admission
Source: BMC Microbiol. 2015 Sep 16;15:181. doi: 10.1186/s12866-015-0514-5 (PMC4573688; doi:10.1186/s12866-015-0514-5)
Supplement: Additional file 5: — Detailed information on five C. difficile negative horses studied via high-throughput amplicon sequencing analysis and compared with C. difficile colonised horses. NSAIDs: nonsteroidal anti-inflammatory drugs. Pen: penicillin; Gen: gentamicin; SXT: trimethoprim/sulfamethoxazole (DOCX 46 kb) [file 12866_2015_514_MOESM5_ESM.docx]

| Clinical history of *C. difficile* negative horses | | | | | | | |
| --- | --- | --- | --- | --- | --- | --- | --- |
| Date of sampling | **Animal identification** | **Age (years)** | **Diagnostic** | **Diarrhoea** | **Hospital stay (days)** | **Antibiotic treatment** | **NSAIDS treatment** |
| 05/10/2013 | 02 | 17 | Colic (displacement of the colon to an abnormal location) | - | 1 | - | Flunixin meglumine Dipyrone |
| 13/11/2013 | 05 | 3 | Colic (recurrent colic due to gastric ulcers) | - | 4 | - | - |
| 19/11/2013 | 06 | 5 | Oesophageal obstruction | - | 1 | - | - |
| 22/11/2013 | 07 | 7 | Wound | - | 2 | Pen-Gen-SXT | Flunixin meglumine  Dipyrone |
| 26/11/2013 | 08 | 7 | Jaw wound | - | 4 | Cefquinome | Dipyrone |
