# Supplementary material for: Faecal microbiota characterisation of horses using 16 rdna barcoded pyrosequencing, and carriage rate of clostridium difficile at hospital admission
Source: BMC Microbiol. 2015 Sep 16;15:181. doi: 10.1186/s12866-015-0514-5 (PMC4573688; doi:10.1186/s12866-015-0514-5)
Supplement: Supplementary file 5 [file 12866_2015_514_MOESM2_ESM.docx]

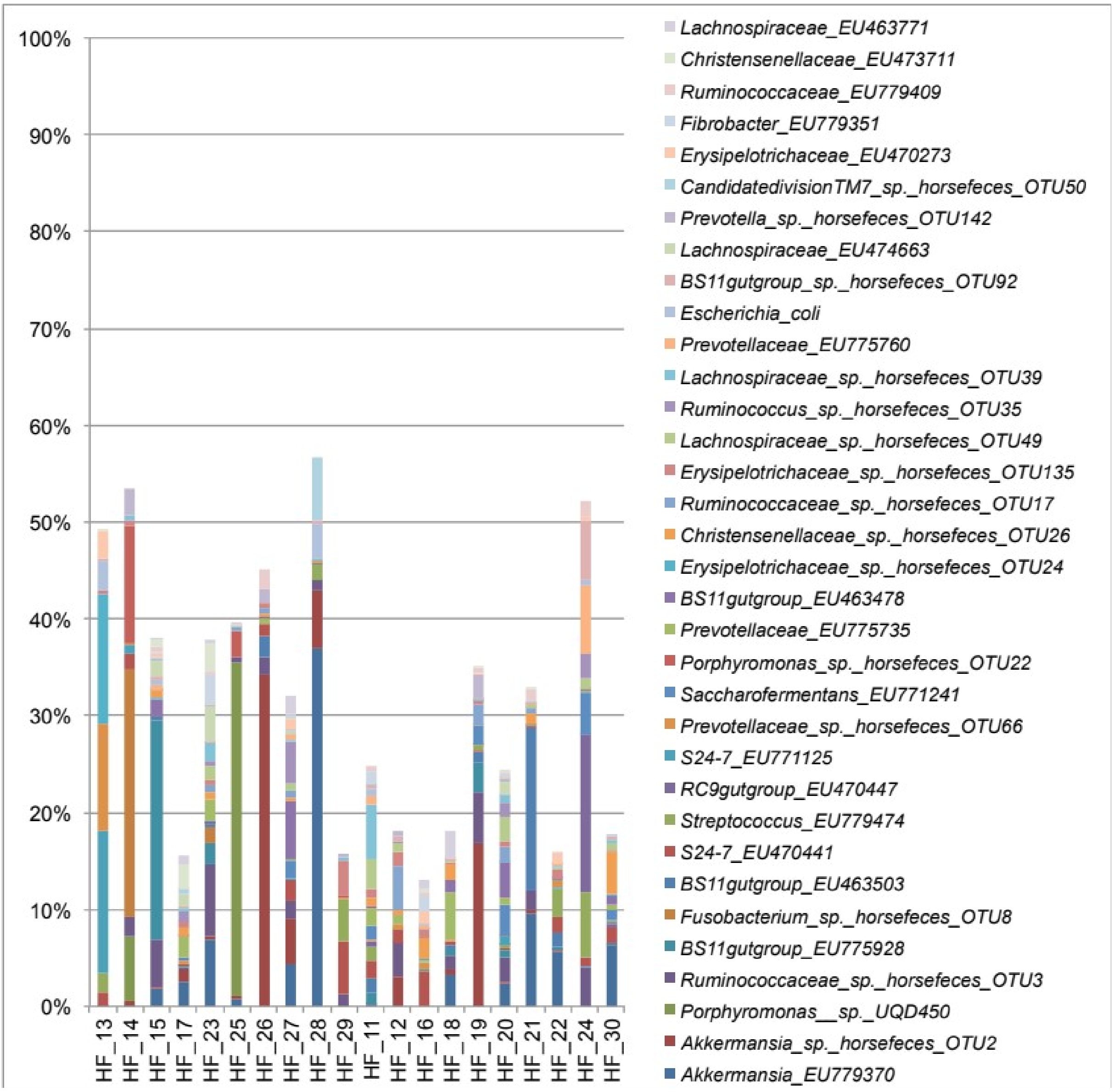
100%
90%
80%
70%
60%
50%
40%
30%
20%
10%
0%
HF_13
HF_14
HF_15
HF_17
HF_23
HF_25
HF_26
HF_27
HF_28
HF_29
HF_11
HF_12
HF_16
HF_18
HF_19
HF_20
HF_21
HF_22
HF_24
HF_30
Lachnospiraceae_EU463771
Christensenellaceae_EU473711
Ruminococcaceae_EU779409
Fibrobacter_EU779351
Erysipelotrichaceae_EU470273
CandidatedivisionTM7_sp._horsefeces_OTU50
Prevotella_sp._horsefeces_OTU142
Lachnospiraceae_EU474663
BS11gutgroup_sp._horsefeces_OTU92
Escherichia_coli
Prevotellaceae_EU775760
Lachnospiraceae_sp._horsefeces_OTU39
Ruminococcus_sp._horsefeces_OTU35
Lachnospiraceae_sp._horsefeces_OTU49
Erysipelotrichaceae_sp._horsefeces_OTU135
Ruminococcaceae_sp._horsefeces_OTU17
Christensenellaceae_sp._horsefeces_OTU26
Erysipelotrichaceae_sp._horsefeces_OTU24
BS11gutgroup_EU463478
Prevotellaceae_EU775735
Porphyromonas_sp._horsefeces_OTU22
Saccharofermentans_EU771241
Prevotellaceae_sp._horsefeces_OTU66
S24-7_EU771125
RC9gutgroup_EU470447
Streptococcus_EU779474
S24-7_EU470441
BS11gutgroup_EU463503
Fusobacterium_sp._horsefeces_OTU8
BS11gutgroup_EU775928
Ruminococcaceae_sp._horsefeces_OTU3
Porphyromonas__sp._UQD450
Akkermansia_sp._horsefeces_OTU2
Akkermansia_EU779370
