# Supplementary material for: Faecal microbiota characterisation of horses using 16 rdna barcoded pyrosequencing, and carriage rate of clostridium difficile at hospital admission
Source: BMC Microbiol. 2015 Sep 16;15:181. doi: 10.1186/s12866-015-0514-5 (PMC4573688; doi:10.1186/s12866-015-0514-5)
Supplement: Supplementary file 6 [file 12866_2015_514_MOESM3_ESM.docx]

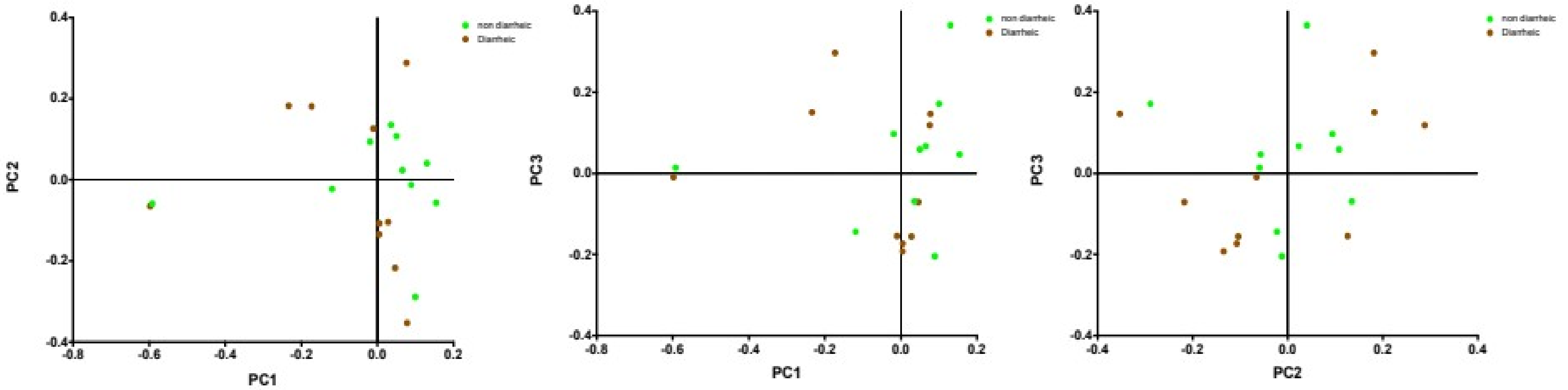

non diarrheic
Diarrheic
PC1
PC2
PC3
0.4
0.2
0.0
-0.2
-0.4
-0.8
-0.6
