# Supplementary material for: Faecal microbiota characterisation of horses using 16 rdna barcoded pyrosequencing, and carriage rate of clostridium difficile at hospital admission
Source: BMC Microbiol. 2015 Sep 16;15:181. doi: 10.1186/s12866-015-0514-5 (PMC4573688; doi:10.1186/s12866-015-0514-5)
Supplement: Supplementary file 7 [file 12866_2015_514_MOESM6_ESM.docx]

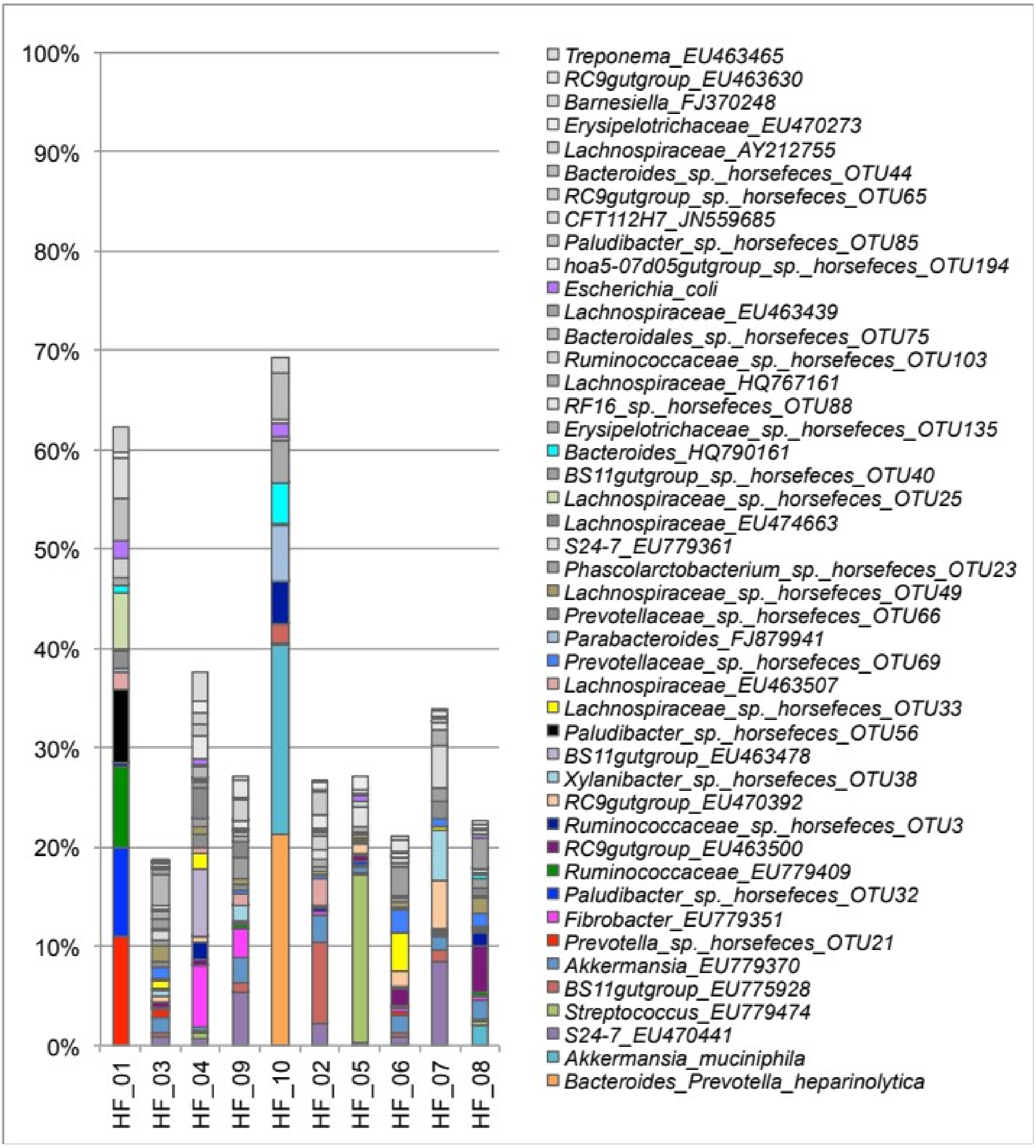
100%
90%
80%
70%
60%
50%
40%
30%
20%
10%
0%
HF_01
HF_03
HF_04
HF_09
HF_10
HF_02
HF_05
HF_06
HF_07
HF_08
Treponema_EU463465
RC9gutgroup_EU463630
Barnesiella_FJ370248
Erysipelotrichaceae_EU470273
Lachnospiraceae_AY212755
Bacteroides_sp._horsefeces_OTU44
RC9gutgroup_sp._horsefeces_OTU65
CFT112H7_JN559685
Paludibacter_sp._horsefeces_OTU85
hoa5-07d05gutgroup_sp._horsefeces_OTU194
Escherichia_coli
Lachnospiraceae_EU463439
Bacteroidales_sp._horsefeces_OTU75
Ruminococcaceae_sp._horsefeces_OTU103
Lachnospiraceae_HQ767161
RF16_sp._horsefeces_OTU88
Erysipelotrichaceae_sp._horsefeces_OTU135
Bacteroides_HQ790161
BS11gutgroup_sp._horsefeces_OTU40
Lachnospiraceae_sp._horsefeces_OTU25
Lachnospiraceae_EU474663
S24-7_EU779361
Phascolarctobacterium_sp._horsefeces_OTU23
Lachnospiraceae_sp._horsefeces_OTU49
Prevotellaceae_sp._horsefeces_OTU66
Parabacteroides_FJ879941
Prevotellaceae_sp._horsefeces_OTU69
Lachnospiraceae_EU463507
Lachnospiraceae_sp._horsefeces_OTU33
Paludibacter_sp._horsefeces_OTU56
BS11gutgroup_EU463478
Xylanibacter_sp._horsefeces_OTU38
RC9gutgroup_EU470392
Ruminococcaceae_sp._horsefeces_OTU3
RC9gutgroup_EU463500
Ruminococcaceae_EU779409
Paludibacter_sp._horsefeces_OTU32
Fibrobacter_EU779351
Prevotella_sp._horsefeces_OTU21
Akkermansia_EU779370
BS11gutgroup_EU775928
Streptococcus_EU779474
S24-7_EU470441
Akkermansia_muciniphila
Bacteroides_Prevotella_heparinolytica
