# Supplementary material for: Faecal microbiota characterisation of horses using 16 rdna barcoded pyrosequencing, and carriage rate of clostridium difficile at hospital admission
Source: BMC Microbiol. 2015 Sep 16;15:181. doi: 10.1186/s12866-015-0514-5 (PMC4573688; doi:10.1186/s12866-015-0514-5)
Supplement: Supplementary file 9 [file 12866_2015_514_MOESM8_ESM.docx]

Statistical differences - genus level - CDIF

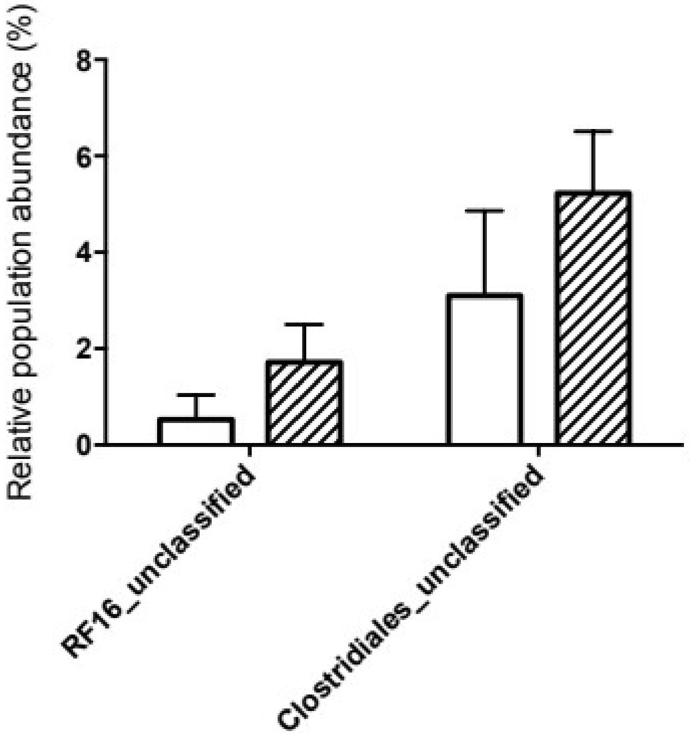

	P value
RF16_unclas:	0.01591304
Clostridiales_	0.0475942
