# Supplementary material for: Faecal microbiota characterisation of horses using 16 rdna barcoded pyrosequencing, and carriage rate of clostridium difficile at hospital admission
Source: BMC Microbiol. 2015 Sep 16;15:181. doi: 10.1186/s12866-015-0514-5 (PMC4573688; doi:10.1186/s12866-015-0514-5)
Supplement: Supplementary file 10 [file 12866_2015_514_MOESM9_ESM.docx]

Statistical differences - genus level - diagnosis groups

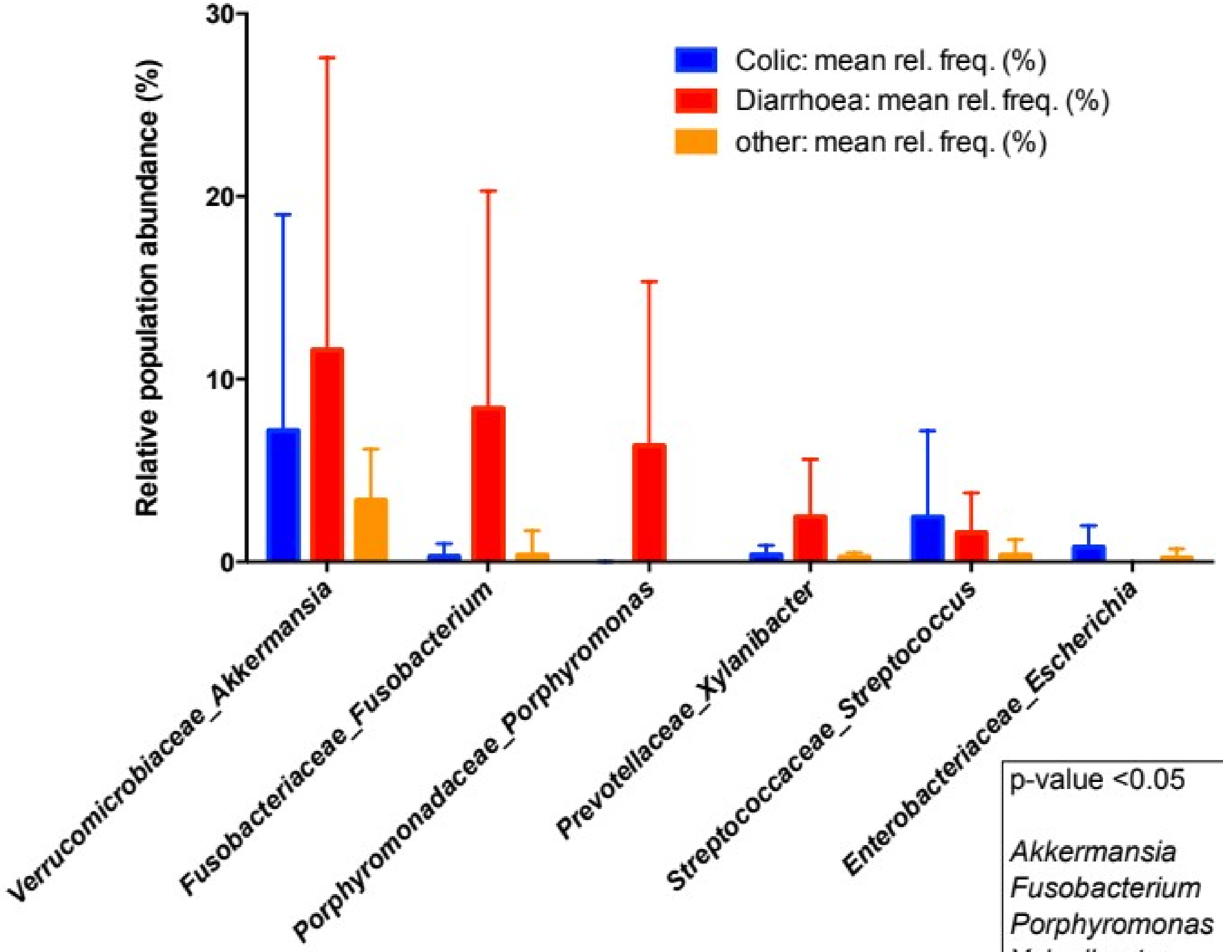

p-value <0.05

	Colic	Diarrhoea	Others
Akkermansia	a	b	c
Fusobacterium	a	b	a
Porphyromonas	a	b	a
Xylanibacter	a	b	a
Streptococcus	a	ab	b
Escherichia	a	b	ab
